# „Gemeinsam stark“ – Preparedness an deutschen Häfen aus Perspektive beteiligter Akteure im Kontext von Infektionsgeschehen

**DOI:** 10.1055/a-2735-5866

**Published:** 2026-01-27

**Authors:** Marie Frese, Julian Bäßler, Matthias Boldt, Martin Dirksen-Fischer, Lena Ehlers, Sarah Nikola Gueye, Volker Harth, Jan Heidrich

**Affiliations:** 1 Zentralinstitut für Arbeitsmedizin und Maritime Medizin, Universitätsklinikum Hamburg-Eppendorf, Hamburg, Germany; 2 Hamburg Port Health Center, Freie und Hansestadt Hamburg Institut für Hygiene und Umwelt, Hamburg, Germany

**Keywords:** Hafen, Infektionsmanagement, Öffentliche Gesundheit, Qualitative Studie, Internationale Gesundheitsvorschriften, Port, Disease Control, Public Health, Qualitative Study, International Health Regulations

## Abstract

**Hintergrund:**

Häfen sind bei Infektionsgeschehen an Bord von Schiffen eine Schnittstelle zur lokalen Bevölkerung. Neben den Gesundheitsbehörden sind weitere Akteure beim Management von Infektionskrankheiten beteiligt. Ein effektiver Ansatz zur Prävention und zum Management von Krankheitsausbrüchen, der alle Beteiligten berücksichtigt, ist daher entscheidend. Diese qualitative Studie innerhalb des Projekts „GESA – Gesunde Häfen, gemeinsam stark“ untersucht die Bedarfe beteiligter Akteure.

**Methoden:**

Im Rahmen von GESA wurden zur Erhebung von Strukturen und Prozessen an fünf großen deutschen Häfen semi-strukturierte Interviews mit Mitarbeitenden der Hafenärztliche Dienste sowie mit Hafenbehörden, Feuerwehren, Terminalbetreibern, Lotsen und weiteren relevanten Akteuren durchgeführt. Hierbei wurden unter anderem auch Optimierungsmöglichkeiten und Handlungsbedarfe erhoben. Die Auswertung der Interviews erfolgte mithilfe der qualitativen Inhaltsanalyse nach Mayring.

**Ergebnisse:**

Insgesamt wurden 34 Interviews durchgeführt, dabei konnten 13 unterschiedliche Akteure abgedeckt werden. Die Bedarfe wurden hauptsächlich in den Bereichen IGV-Notfallplanung, interdisziplinärer Austausch, Digitalisierung, Schnittstellen und Informationsbedarf im Einsatzgeschehen sowie Training und Fortbildung geäußert.

**Schlussfolgerung:**

Das Projekt GESA liefert einen wichtigen Beitrag zur „Port Preparedness“, da die erhobenen Bedarfe direkt in die Entwicklung eines idealtypischen Prozesses für das Infektionsmanagement an deutschen Häfen einfließen. Zudem werden die Ergebnisse bei der Erstellung eines Schulungskonzept für den Öffentlichen Gesundheitsdienst berücksichtigt.

## Abstract (en)

**Background:**

Ports are the first interface with the local population in the event of infections on board ships. In addition to the health authorities, other stakeholders are also involved in the management of disease outbreaks or are affected by them through their activities at the port. An effective approach to outbreak prevention and management that takes all stakeholders into account is therefore crucial. This qualitative sub-study of the ‘GESA – Healthy Ports, Strong Together’ project analyses the needs of the stakeholders involved.

**Methods:**

As part of the GESA study, qualitative interviews were conducted with port medical services, port authorities, fire services, terminal operators, pilots and other relevant stakeholders in order to record structures and processes in five major German ports. Among other things, optimisation opportunities and fields of action were identified. The interviews were analyzed using a qualitative content analysis according to Mayring.

**Results:**

A total of 34 interviews were conducted, covering 13 different stakeholders. The needs were primarily expressed in the areas of emergency planning, interdisciplinary exchange, digitalisation, interfaces and information requirements in operations as well as training and further education.

**Conclusion:**

The GESA project makes an important contribution to disease prevention and management at ports, as the needs identified are directly incorporated into the development of an idealised process for infection management in German ports. The results will also be taken into account in the development of a training concept for the public health service.

## Einleitung

Im maritimen Sektor besteht ein erhöhtes Risiko für die Verbreitung von Infektionskrankheiten, da Schiffe häufig zwischen Regionen mit unterschiedlichen Gesundheitsstandards und Krankheitsprofilen verkehren. Der enge Kontakt zwischen internationalen Besatzungen und Passagieren, sowie die spezifischen Bedingungen an Bord können die Ausbreitung von Infektionen begünstigen 1 2 . Gleichzeitig erhöht sich das Risiko für die lokale Bevölkerung, was den öffentlichen Gesundheitsdienst vor erhebliche Herausforderungen stellt 3 . Um die Übertragung von Krankheiten an internationalen Grenzübergangsstellen (Points of Entry) zu minimieren und eine schnelle, koordinierte Reaktion zu gewährleisten, hat die Weltgesundheitsorganisation (WHO) mit den Internationalen Gesundheitsvorschriften (IGV) weltweit gültige Standards festgelegt 4 . In Deutschland sind fünf Häfen (Bremen/Bremerhaven, Hamburg, Kiel, Rostock, Wilhelmshaven) für die Umsetzung der IGV benannt. Diese Häfen müssen bestimmte Ausstattungen und Kernkapazitäten vorhalten, um adäquat auf Gesundheitsgefahren reagieren zu können 5 . Wenn an Bord von Schiffen Infektionsgeschehen auftreten, ist eine Vielzahl von Akteuren landseitig involviert. Dies betrifft sowohl diejenigen, die routinemäßig ihrer Tätigkeit im Hafen oder an Bord nachgehen, als auch jene, die speziell zur Bewältigung solcher Fälle hinzugezogen werden. Eine enge Zusammenarbeit aller beteiligten Akteure, darunter Hafenärztliche Dienste, Hafenbehörden, Feuerwehren, Lotsen, Wasserschutz- und Bundespolizei, ist für eine koordinierte und schnelle Reaktion unerlässlich 6 .

### Zielsetzung und Forschungsfrage

Ziel des Verbundforschungsprojektes „Gesunde Häfen, Gemeinsam Stark (GESA)“ ist die Entwicklung einer standortübergreifenden koordinierten Strategie, die auf die Bewältigung infektiologischer Gefahrenlagen abzielt 7 . Im Rahmen der hier beschriebenen qualitativen Teilstudie erfolgte eine Bedarfsanalyse zu Prozessen, Herausforderungen und Optimierungsmöglichkeiten der jeweiligen Akteure. Als Methodik dienen qualitative Interviews mit Mitarbeitenden relevanter Berufsgruppen, um präzise Einblicke in die Erfahrungen und Bedarfe der unterschiedlichen Arbeitswelten zu erhalten.

## Methodik

### Setting und Auswahl der Studienpopulation

Die Auswahl der Studienpopulation an den fünf IGV-Häfen erfolgte unter Berücksichtigung ihrer direkten Betroffenheit von Infektionsgeschehen an Bord bzw. ihrer aktiven Einbindung in derartige Situationen. Die Kontaktaufnahme mit relevanten Akteuren erfolgte in Zusammenarbeit mit den Hafenärztlichen Diensten. Gemäß der zuständigen Ethikkommission der Ärztekammer Hamburg ist für diese Studie kein Ethikvotum erforderlich (Kennzeichen 2023-300340-WF).

### Datenerhebung

Als Erhebungsinstrument wurden zwei semi-strukturierte Leitfäden entwickelt, jeweils einer für die hafenärztlichen Dienste sowie einer für die weiteren Akteure. Sie beinhalteten Fragen zu spezifischen Tätigkeiten und Einsatzabläufen, Schnittstellen zwischen den Akteuren, Erfahrungen aus vergangenen Infektionsausbrüchen, Handlungsbedarfen sowie Übungs- und Schulungspraxis. Die Interviews erfolgten auf freiwilliger Basis und nach Unterzeichnung einer Einverständniserklärung, bei der die Teilnehmenden über Zielsetzung und datenschutzrechtliche Aspekte informiert wurden. Von September bis Dezember 2023 fanden die meisten Interviews als Face-to-Face-Gespräche direkt vor Ort an den jeweiligen Arbeitsstätten statt. Das Studienteam hielt sich hierfür jeweils zwei bis drei Tage an den Standorten auf und konnte gleichzeitig Eindrücke der Hafenanlagen sammeln. Einzelne Interviews wurden über WebEx durchgeführt. Die Gespräche wurden mit einem Diktiergerät aufgezeichnet, anschließend mit der Software Amberscript transkribiert, manuell nachbearbeitet und anonymisiert.

### Datenauswertung

Die Auswertung erfolgte mit MaxQDA24 nach der strukturierenden Inhaltsanalyse gemäß Mayring 8 . Ein deduktives Kategoriensystem mit vier Oberthemen und 19 Unterthemen wurde literaturbasiert entwickelt. Initial wurden zwei Transkripte kodiert, um das System zu prüfen und induktiv zu erweitern. Anschließend wurden die restlichen Transkripte kodiert und das System kontinuierlich angepasst. Muster und Zusammenhänge in den kodierten Daten wurden analysiert und im Kontext der Forschungsfrage diskutiert.

## Ergebnisse

### Charakterisierung der Studienpopulation

Insgesamt wurden 34 Interviews durchgeführt. Befragt wurden Mitarbeitende aus 13 verschiedenen relevanten Akteursgruppen, die Teilnahme variierte je nach Standort. Mitarbeitende des Hafenärztlichen Dienstes, der Hafenbehörde und der Feuerwehr konnten an allen fünf Standorten befragt werden. Die Anzahl der befragten Akteure sowie Tätigkeiten und Zuständigkeiten, insbesondere im Zusammenhang mit Infektionsereignissen, sind in Tab. 1 dargestellt.

**Table:** **Tab. 1** Anzahl befragter Akteure und jeweilige Tätigkeiten und Zuständigkeiten im Kontext von Infektionsereignissen.

Akteur	n	Zuständigkeiten
Hafenärztlicher Dienst	5	Ermittlung von Erkrankten und Kontaktpersonen Festlegung weiterer Maßnahmen (u.A. Diagnostik, Quarantäne, Isolation Verdachtsfälle) Zusammenarbeit mit dem ÖGD außerhalb des Hafens
Hafenbehörde / Hafenkapitän	6	Regelung des ruhenden/fließenden Verkehrs
Kanalsteurer	1	Schiffsführungen durch den Nord-Ostseekanal
Lotsen	4	Beratung von Schiffsführungen beim Anlaufen in den Hafen
Agentur	2	Vermittlung zwischen Akteuren und Schiffen
Reederei	1	Umsetzung von Maßnahmen & Informationsweitergabe an Bord
Terminalbetreiber	3	Terminalsicherheit- und Koordination Bereitstellung von Personal vor Ort (Security, Reinigung, etc.)
Feuerwehr	5	Ggfls. Koordination eines gemeinsamen Notfallmanagements Medizinische Versorgung und Transport von Erkrankten Durchführung von Infektionstransporten
Bundespolizei	1	Kontrolle des grenzüberschreitenden Personen- und Fahrzeugverkehrs Erteilung von Passierscheinen
Wasserschutzpolizei	2	Verhütung und Verfolgung von Straftaten/Ordnungswidrigkeiten Kontrolle von Papieren, Zeugnissen und Dokumenten auf Schiffen Freihalten von Rettungswegen und Absperrung von Einsatzräumen
Zollamt	1	Einklarierung der Schiffe Kontrolle von Papieren, Zeugnissen und Dokumenten auf Schiffen
Havariekommando	1	Medizinische Versorgung auf hoher See (Maritime Incident Response Groups) Ggfls. Koordination eines gemeinsamen Notfallmanagements
Seemannsmission	2	Psychosoziale Notfallversorgung (PSNV) von Crewmitgliedern Organisation von Unterbringungsmöglichkeiten

### Bedarfe im Kontext von Infektionsgeschehen

**Abb. 1:**
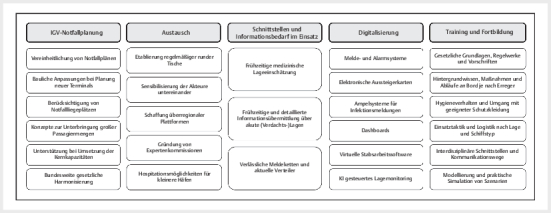
Übersicht zu den verschiedenen identifizierten Bedarfen.

Es wurden Bedarfe in fünf Kategorien identifiziert, die im Folgenden akteursübergreifend erläutert werden. Diese umfassen Vorschläge und Anmerkungen zur Optimierung bestehender Prozesse und Strukturen. Die Kategorien umfassen IGV-Notfallplanung, interdisziplinärer Austausch, Schnittstellen und Informationsbedarf im Einsatz, Digitalisierung sowie Training und Fortbildung und werden im Folgenden beschrieben, sie sind ebenfalls in Abb. 1 abgebildet. Die entsprechenden Zitate befinden sich im **Online-Supplement** .

#### IGV-Notfallplanung

Im Bereich der IGV-Notfallplanung und Hafeninfrastruktur wurden verschiedene Empfehlungen ausgesprochen, um besser auf zukünftige Herausforderungen und Krisensituationen vorbereitet zu sein. Dazu gehört die Vereinheitlichung von Notfallplänen durch die Erstellung eines generischen Plans, der von den Häfen angepasst werden kann und wichtige Prozesse sowie Strukturen wie Kommunikationswege und Hafengrundrisse berücksichtigt. Es wurde auch vorgeschlagen, Infektionsschutzmaßnahmen bereits bei der Planung neuer Hafenbauten, insbesondere bei Großterminals, zu integrieren. Die Vorhaltung von Notfallliegeplätzen für Infektionsfälle und die Entwicklung von Konzepten zur Notunterbringung großer Passagiermengen durch temporäre Infrastrukturen wie Zelte wurden ebenfalls thematisiert. Zudem wurde empfohlen, einen zentralen Lagerort für von der Bundesrepublik bereitgestelltes Material zu schaffen, um eine schnelle Lieferung an alle IGV-Häfen zu gewährleisten. Es besteht Unterstützungsbedarf bei der Umsetzung der Empfehlungen des Robert Koch-Instituts zu den IGV-Kernkapazitäten, insbesondere im Bereich der baulichen Anforderungen. Eine detaillierte Handreichung oder Prioritätenliste für die Umsetzung der Maßnahmen wird benötigt. Viele Befragte wünschten sich zudem eine bundesweite Harmonisierung der Gesetze und Vorgaben, um einheitliche Standards für alle Häfen zu schaffen und den überregionalen Austausch und die eigene Arbeit zu erleichtern.

#### Austausch

Um das Management von Infektionsereignissen zu verbessern, wurden von verschiedenen Akteuren Vorschläge für einen kontinuierlichen und nachhaltigen interdisziplinären Austausch unterbreitet. Regelmäßige Treffen, wie „runde Tische“, wurden empfohlen, um den Erfahrungs- und Wissensaustausch zu intensivieren, Notfallpläne zu aktualisieren und auf personelle Fluktuationen zu reagieren. Die Sensibilisierung relevanter Akteure wurde ebenfalls betont. Ein regelmäßiger Austausch über Erfahrungswerte und bewährte Verfahren soll das gegenseitige Verständnis fördern und die eigenen Prozesse verbessern. Zudem wurde der Aufbau einer überregionalen Kommunikationsplattform zwischen Häfen und Bundesländern vorgeschlagen, um von Fallanalysen und Erfahrungen anderer Standorte zu profitieren. Eine Expertenkommission, die sich jährlich trifft und Empfehlungen für die Einsatzplanung entwickelt, wurde ebenfalls angeregt. Hospitationsmöglichkeiten wurden als sinnvoll erachtet, damit Gesundheitsbehörden aus kleineren Häfen von den Erfahrungen größerer profitieren können.

#### Schnittstellen und Informationsbedarf im Einsatz

Um die Kommunikation bei Infektionsfällen zu optimieren und eine schnelle, koordinierte Reaktion zu gewährleisten, wurden von den befragten Personen mehrere Empfehlungen ausgesprochen. Die Bedeutung einer frühzeitigen und detaillierten Informationsübermittlung über die Lage an Bord wurde mehrfach betont. Insbesondere Akteure, die an Bord arbeiten, wünschten sich frühzeitige Informationen über die Isolation infizierter Personen und deren Arbeitsbereiche, um ihren Eigenschutz zu gewährleisten. Eine frühzeitige medizinische Lageeinschätzung an Bord wurde ebenfalls empfohlen, um mögliche Risiken bereits vor dem Erreichen des Hafens zu erkennen und entsprechend zu reagieren. Die Befragten hoben die Notwendigkeit verlässlicher Meldeketten und aktueller Verteiler hervor. Es wurde vorgeschlagen, einen regelmäßig aktualisierten und verbindlichen Verteiler zu pflegen, um im Falle einer Infektionslage schnell die relevanten Akteure zu erreichen und eine zügige Kommunikation zu ermöglichen. Zudem wurde die Einrichtung von durchgängig erreichbaren, zentralen Anlaufstellen auch an kleineren Häfen empfohlen.

#### Digitalisierung

Im Bereich der Digitalisierung wurden verschiedene Vorschläge gemacht, um die Effizienz, Kommunikation und Koordination im Notfallmanagement zu verbessern. Einige Befragte empfahlen die Implementierung digitaler Melde- und Alarmsysteme, die im Falle eines Infektionsereignisses alle relevanten Akteure automatisch benachrichtigen und einen schnellen Überblick über die Lage ermöglichen. Dies sollte durch die Digitalisierung der Meldewege ergänzt werden, um beispielsweise die Infektionsmeldungen gemäß §12 IfSG effizienter zu gestalten. Auch die Digitalisierung von Aussteigerkarten zur Kontaktnachverfolgung wurde vorgeschlagen, um die Verwaltung zu vereinfachen und die Bearbeitung zu beschleunigen. Ein weiterer Vorschlag war die Einführung eines Ampelsystems für Infektionsmeldungen, das Infektionsrisiken schnell erfassbar macht, vorausgesetzt, die Schiffe liefern valide Daten. Die Einrichtung eines Dashboards, das Krankheitsfälle an Bord erfasst und diese Daten in Echtzeit an die zuständigen Gesundheitsbehörden übermittelt, wurde ebenfalls als nützlich erachtet. Ein weiterer Vorschlag betraf den Einsatz von Stabsarbeitssoftware, die es ermöglicht, alle Beteiligten zu vernetzen, Informationen zentral hochzuladen und effizient zu teilen. Dies wird als erhebliche Verbesserung im Vergleich zu klassischen Methoden wie Telefon oder E-Mail wahrgenommen, da die Zusammenarbeit strukturierter und transparenter gestaltet wird. Zudem wurde die Entwicklung eines mittels Künstlicher Intelligenz (KI) gesteuerten Lagemonitorings genannt, das die globale Krankheitslage einbezieht, um frühzeitig und lagegerecht auf internationale Bedrohungen reagieren zu können.

#### Training und Fortbildung

Es wurden Schulungen zur Risikoeinschätzung von Infektionen und Übertragungswegen sowie zu exotischen Krankheiten und wiederkehrenden Erregern wie Tuberkulose und Hepatitis vorgeschlagen. Ergänzend sollen Übersichtshilfen zu potenziellen Infektionen erstellt werden, um Unsicherheiten zu reduzieren. Zudem wurde empfohlen, Schulungen zum richtigen Einsatz persönlicher Schutzausrüstung, wie Schutzanzügen und Masken, durchzuführen. Weitere Schulungen sollten IGV-Basiswissen und Hafenresilienz – die Fähigkeit eines Hafens, sich von Störungen und Krisen zu erholen und seine Funktionsfähigkeit aufrechtzuerhalten – umfassen. Ein weiteres Schulungsthema umfasst dem Bereich der Infektionsprävention und -kontrolle, etwa durch Szenariosimulationen, Quarantänevorkehrungen und spezifisches Wissen über die besonderen Anforderungen von Kreuzfahrtschiffen. Darüber hinaus wurde die Durchführung regelmäßiger Planspiele und Vollübungen vor Ort genannt, um Abläufe praxisnah zu trainieren. Dabei wurden realistische Szenarien mit Faktoren wie Erregertypen und Passagierbewegungen als wichtig erachtet. Die Stärkung von Kommunikation und interdisziplinärer Zusammenarbeit durch Übungen wurde ebenfalls als zentral angesehen.

## Diskussion

Unsere Untersuchung mittels semi-strukturierter Interviews mit relevanten Akteuren zeigt, dass im Kontext von Infektionsgeschehen am Hafen vielfältige Bedarfe bestehen. Die Berücksichtigung dieser Bedarfe ist von entscheidender Bedeutung für die erfolgreiche Bewältigung von Krisensituationen. Daraus lassen sich Anregungen zur ganzheitlichen Stärkung der Preparedness an deutschen Häfen ableiten.

Die COVID-19-Pandemie hat gezeigt, dass föderale Unterschiede in der Gesetzgebung zu Unsicherheiten und Schwierigkeiten führten. Eine gesetzliche Harmonisierung ist daher entscheidend, um diese Probleme zu minimieren. Einheitliche Gesetze verbessern die Effizienz und Kohärenz der Maßnahmen und stärken die Compliance der Schifffahrt. Klare Regelungen ermöglichen es Schifffahrtsunternehmen, Maßnahmen besser zu verstehen und umzusetzen, was die Sicherheit und Zuverlässigkeit im maritimen Sektor erhöht. Gleichzeitig sind die Vorhaltung und regelmäßige Aktualisierung von Notfallplänen essenziell. Eine Vereinheitlichung der Struktur dieser Pläne stellt sicher, dass alle relevanten Inhalte berücksichtigt werden.

Der Fokus auf präventive Maßnahmen ist entscheidend, um Unsicherheiten über Krankheitsübertragungen zu verringern und die Resilienz von Mitarbeitenden und Organisationen zu stärken. Regelmäßige Trainings und Schulungen sind notwendig, um Wissen aktuell zu halten und neue Entwicklungen einzubeziehen. Vollübungen zum Training von Schnittstellen und Abläufen wurden von den Befragten positiv bewertet, sind jedoch ressourcen- und kostenintensiv. Kleinere Formate wie Planspiele bieten niedrigschwellige Alternativen. Digitale Übungsformate haben sich als effektive Alternative zu Präsenzübungen bewährt, wie die virtuellen Stabsrahmenübungen im ARMIHN-Projekt 2021 in Hamburg gezeigt haben 9 . Kontinuierliche Kommunikation und Austausch zwischen den Akteuren sind ebenfalls entscheidend, auch unabhängig von Einsatzlagen. Eine lückenlose Meldekette ist unerlässlich, um alle Akteure frühzeitig informieren und warnen zu können. Dies gilt insbesondere für Lotsen, die als Erste an Bord gehen und sich entsprechend schützen müssen. Kommunikationsbarrieren sollten daher durch Maßnahmen wie Runde Tische und Kommunikationsübungen abgebaut werden, um die Sensibilisierung für die Tätigkeiten und Zuständigkeiten der beteiligten Akteure zu steigern. Die Stärkung von Netzwerken zum gegenseitigen Austausch und die Nutzung digitaler Systeme für eine Vereinfachung der Abläufe im Flug- und Schiffverkehr wurden bereits im Kontext der COVID-19-Pandemie diskutiert 3 .

Ein innovatives und vielversprechendes Feld eröffnet sich zudem durch die Integration von Künstlicher Intelligenz (KI) und algorithmisch gesteuerten Systemen zur Lageeinschätzung und Bewertung von Infektionsgeschehen im maritimen Transportsektor. Diese Technologien ermöglichen es, globale Datensätze umfassend zu analysieren und einzubeziehen, was zu einem frühzeitigen Erkennen von Gefahren und präziseren Risikobewertungen führt. Ansätze mit KI gibt es bereits im Bereich der maritimen Sicherheit und Risikomanagement 10 .

### Limitationen und Stärken

Im interdisziplinären Projekt GESA wurden erstmals die Prozesse und Bedürfnisse verschiedener Akteure an den fünf IGV-Häfen umfassend untersucht, um unterschiedliche Perspektiven zu berücksichtigen. Durch die Unterstützung der Hafenärztlichen Dienste konnte ein breites Spektrum der Akteurslandschaft abgedeckt werden. Es besteht jedoch die Möglichkeit, dass einzelne Gruppen unterrepräsentiert sind, da die Teilnahme je nach Standort variierte. Die Durchführung von Runden Tischen mit verschiedenen Akteuren ermöglichte eine umfassende Einordnung der Untersuchungsergebnisse und förderte den Austausch zwischen den Beteiligten.

### Ausblick

Die ermittelten Bedarfe fließen in die Entwicklung eines idealtypischen generischen Prozesses und Notfallplans zum Infektionsmanagement an deutschen IGV-Häfen ein, welcher im Rahmen des Projektes erstellt und den Häfen zur Verfügung gestellt wird. Die Ergebnisse der Studie und eine Berücksichtigung der Bedarfe aller beteiligten Akteure ermöglichen es, Häfen nicht nur besser auf Ernstfälle vorzubereiten, sondern auch flexibel und effektiv auf unterschiedliche Infektionsgeschehen zu reagieren. Eine koordinierte und gut durchdachte Strategie zur Infektionsprävention und Bekämpfung im maritimen Bereich unterstützt somit nicht nur die Gesundheit und Sicherheit der direkt Betroffenen, sondern leistet auch einen wichtigen Beitrag zum allgemeinen öffentlichen Gesundheitsschutz.

## Zusätzliches Material

Zusätzliches Material Zusätzliches Material
