# Supplementary material for: “Strong together”: Preparedness in German ports from the perspective of the stakeholders involved in the context of infection events
Source: Gesundheitswesen. 2026 Jan 27;88(7):475–80. [Article in German] doi: 10.1055/a-2735-5866 (PMC13345682; doi:10.1055/a-2735-5866)
Supplement: Supplementary file 1 — Zusätzliches Material [file 10-1055-a-2735-5866-gesu-2025-04-2260-oa.pdf]

Supplement: Ausgewählte Aussagen aus den Interviews nach Kategorien

Oberkategorie IGV-Notfallplanung	**Standardisierte Notfallpläne**	*„[…] ob man nicht vielleicht so eine unabhängig von, also unter der Berücksichtigung der Verwaltungsebenen, Beispielprozesse oder so Beispielverfahren entwickeln kann, dass man den Häfen das mit an die Hand gibt und sagt: Guckt mal, ihr müsst, weil alle Häfen müssen ja den Plan machen, ihr müsst ja den Plan machen, das hier ist ein Beispielplan, der auf 90 Prozent eigentlich alle zutrifft, ändert den noch ein bisschen ab, dass er für euch passt, so in die Richtung vielleicht und dann auch halt für die Kommunikation. Wer muss am besten mit wem in Kommunikation stehen, so in die Richtung vielleicht?" (Hafenbetreiber)*<br><br>*„Die Hafenärztlichen Dienste, die Hafenbetreiber […], Hafenbehörden. Das wären erst mal die drei wichtigsten, würde ich mal sagen, dass die sich mal treffen, Erfahrungen austauschen. Ihre Pläne, ist ja kein Geheimnis, kein Hexenwerk, kein Geheimplan, dass sie mal ihre Pläne vergleichen vielleicht sogar, die Örtlichkeiten sind überall anders und verschieden, aber dass man das, was man vereinheitlichen könnte, dann auch vereinheitlicht." (Hafenbehörde)*
	**Frühzeitige bauliche Anpassungen und Vorhaltung von Notliegeplätzen**	*„Genau und wo man auch was in der Hand hat, wenn zum Beispiel so ein Großterminal neu gebaut wird, dass man sagt hier aber das und das, also dass man so einen gewissen Standard schafft der Länderübergreifend ist, also dass man sagt, ein Kreuzfahrthafen muss das und das und das haben so in der Art." (Hafenärztlicher Dienst)*<br><br>*„Wo wir natürlich Probleme haben, insgesamt aber den Hafen betreffend, ist Liegeplätze dann tatsächlich für das Schiff zu finden, da haben wir generell in den letzten Jahren immer mehr Schwierigkeiten. Was Liegeplätze betrifft, sei es aufgrund der Tiefe oder auch von den Kaianlagen, die gesperrt werden müssen, aufgrund ihres Zustandes. Da müssen wir schauen, wie wir dann in den nächsten Jahren, Jahrzehnten damit umgehen, und dass wir dann eben halt auch weiterhin Plätze vorhalten für genau diese genannten Fälle. Das ist so ein bisschen, was so die Herausforderung für uns jetzt persönlich hier dann darstellt." (Hafenbehörde)*
	**Materialvorhaltung zur Notunterbringung großer Passagiermengen**	*„Mit 20 Leuten kann man noch relativ gut umgehen, ne typische Besatzung eines Containerschiffes oder eines Carcarriers, aber was mache ich tatsächlich, wenn ich jetzt mich mit einem Kreuzfahrtschiff konfrontiert sehe? Und da sehe ich natürlich auch das größte Risiko eines tatsächlichen Ausbruchs. Es müssen ja nicht immer gleich die tödlichsten Seuchen sein, das reicht ja schon, sage ich mal, eine Durchfallerkrankung, oder wie auch immer. Bei 6000 Passagieren ist das echt eine Hausnummer, insofern sie dann vielleicht auch noch irgendwie in irgendeiner Art und Weise ansteckend sein sollte und man darüber nachdenken muss, wo bringe ich jetzt diese Leute irgendwie unter? […] da würde ich mir natürlich wünschen, dass es, sag ich mal, vielleicht auch vom Bund etwas entsprechendes vorgehalten wird, seien es nur irgendwelche Zelte oder wie auch immer, die schnell auf einer freien Fläche aufgebaut werden können, wo man entsprechend die Leute unterbringen könnte. […] Flächen können im Bereich von Containerhäfen immer schnell hergestellt werden dafür, also so etwas würde ich mir tatsächlich wünschen, weil ich mir sage, es steht halt einfach nicht zur Verfügung." (Hafenbehörde)*<br><br>*„Also das ist, das ist, glaube ich, erst mal so, im Großen und Ganzen das, was wir benötigen, um schnell auch auf so etwas reagieren zu können. Ich sag mal, die IGV-Häfen, sind ja alle räumlich relativ nahe beieinander. Wenn der Bund jetzt irgendetwas zur Verfügung stellen würde, müsste er ist ja nicht in München lagern, sondern er würde es wahrscheinlich irgendwo in Hamburg lagern, weil Hamburg recht zentral liegt und von dort aus relativ schnell, eigentlich innerhalb von zwei Stunden, in alle IGV-Häfen geliefert werden könnte." (Hafenbehörde)*
	**Umsetzung der RKI Empfehlungen zu Kernkapazitäten**	*„Also ich fände es gut, wenn man mal die Kernkapazitäten beleuchtet und eine Prioritätenliste macht, was zuerst da sein soll, was wirklich eine wichtige Sache ist und was dann eher nice to have ist oder vielleicht sogar nicht unbedingt umgesetzt werden braucht, ja. Das ist nämlich, ich habe nämlich bei dieser joint external evaluation gesehen, dass das gar kein Thema war bisher hier, was man alles vorhalten muss, außer den personellen Kapazitäten. Das war natürlich schon immer Thema, aber so die ganzen baulichen Sachen und dass man sich darüber mal im klaren ist und vielleicht auch einen gemeinsamen Konsens findet, was man wirklich braucht, das würde ich jetzt sehr begrüßen." (Hafenärztlicher Dienst)*<br><br>*„Okay, es ist halt so, also wir kennen diese Empfehlung vom RKI ja auch! Es ist halt trotzdem selbst. Da ist es schwer, irgendwie rauszulesen als Laie trotzdem, was bedeutet das jetzt? Und ich hatte auch schon einmal beim RKI angefragt aber die Antwort war auch ein bisschen, naja spärlich. Da wo im Prinzip das nochmal zitiert, was ich schon gelesen habe. So nach dem Motto: "steht ja da." Ja aber sonst im Plan, den man so...wie gesagt so eine Hülle im Prinzip." (Hafenbetreiber)*<br><br>*„Es ist eben, […], dass wir eben die mangelnde Regelung, also die Durchführungsverordnung für Mecklenburg-Vorpommern existiert noch nicht, die hat noch nie existiert, und man will eben die jetzt schnellstmöglich schreiben, von der Landesseite her. Aber nach der Lesart aller Beteiligten, die sich in Rostock zu dem Thema sehr regelmäßig verständigen, ich wiederhole das ja jetzt auch, Gesundheitsamt, Hafenärztlicher Dienst, Rostock Port und das Hafenamt, sind wir uns einig, dass wir angewiesen sind auf die Bestimmung der Kernkompetenzen. Und die Bestimmung der Kernkompetenzen, das muss am Ende das Land machen, und die sind eben ausstehend. Und solange die ausstehend sind, kann eigentlich keiner was machen, aus meiner Sicht. Ich bin in der Runde, die ich eben genannt habe, eigentlich der größte Verfechter dieses Themas, weil wir sind nun mal auf die Weisung der uns übergeordneten Stelle, und das ist in dem Fall das Land, angewiesen, und wenn das Land an der Stelle seine Weisung uns gegenüber nicht erteilt, dann können wir es auch nicht umsetzen." (Feuerwehr)*
	**Gesetzliche Harmonisierung**	*„Also, so etwas könnte ich mir vorstellen und natürlich eine einheitliche Gesetzgebung dazu da vor Ort, das ist glaub ich ganz ganz wichtig, dass wir so etwas zur Verfügung stellen können. Dann wäre es für alle einfacher, vor allen Dingen, weil auch der direkte Austausch auch unter den Hafenbehörden einfach würde." (Hafenbehörde)*
Oberkategorie Austausch	**Lokaler Austausch zwischen Akteuren**	*„Ja, man müsste eigentlich, wir sollten uns alle regelmäßiger zu diesen Themen tatsächlich austauschen. Mit regelmäßig meine ich, dass man sich wenigstens einmal im Jahr zu so einem Thema mal trifft. Weil das ist, ehrlich gesagt, nicht passiert. So keiner ist da traurig drum. Aber letztendlich, wenn dann mal so ein Dampfer da liegt, dann weiß ich jetzt schon, da gucken sich alle an, und alle werden hier, irgendwie fragen ist der jetzt schon genehmigt der Notfallplan und reingucken, und was geht mit dem, und dann gucken wir mal so." (Terminalbetreiber)*<br><br>*„Also wir pflegen da schon Kontakt auch irgendwo, wir unterhalten uns miteinander. Es gibt auch gewisse Situationen, wo wir auch telefonieren mal miteinander, wo auch Nachfragen kommen: Hey, wisst ihr dazu was, oder wie ist das eigentlich? Also, es ist schon ein interagierender Prozess, der auch im Fluss ist. An der einen oder anderen Stelle könnte er sicherlich vielleicht auch besser sein oder vielleicht auch mal alle an einem runden Tisch sitzen. Das hat es glaube ich 2018 hier mal gegeben, da gab es eine Katastrophenschutzübungen. Da saßen alle in einem runden Tisch, aber das ist natürlich auch schon ein paar Jahre her. Und Fluktuation gibt es da auch." (Hafenärztlicher Dienst)*<br><br>*„Also, ich glaube, es würde Sinn machen, wenn man sich noch mehr mit denen zusammensetzt oder auch einfach mal mit der Wasserschutzpolizei zusammensetzt oder vielleicht auch mit dem hafenärztlichen Dienst. Das ist so ein bisschen über die Corona Zeit auch einfach eingeschlafen, aber jeder hat so seinen Arbeitsweg gefunden. Jetzt also gerade bei uns, jetzt mussten wir gucken, wie läuft diese Saison, was, was entwickelt sich so über die Zeit, wo ja jetzt einigermaßen wieder Normalität herrscht? Vielleicht ändert sich das auch fürs nächste Jahr wieder, dass man dann einmal guckt, okay, was sind eigentlich so eure Erfahrungswerte, und irgendwie ist das ganz spannend, wenn man dann auch mal jemanden vor Ort trifft und sich mal austauscht, und dann ist es ach so, ach, deswegen macht ihr das immer so: Ach, das wusste ich ja gar nicht so! […] Also, das würde uns helfen, wenn wir da mal uns einen ganz großen Tisch setzen mit allen irgendwie." (Agentur)*<br><br>*„Das heißt, wenn wir einen Fall haben oder einen Anlass haben, müssen diese Akteure Verkehrszentrale, Hafenärztlicher Dienst, Lotsenbrüderschaft - das sind die, die für mich wichtig sind - die müssen sich mal treffen und darüber mal sprechen, damit man ein Gesicht hat und weiß, da gab es ja auch noch Lotsen, die müssen da ja an Bord gehen und das klappt dann beim nächsten Mal besser. […] Dann entsteht ein Gefühl dafür, wen könnte das eigentlich noch interessieren, dass auf dem Schiff irgendwas nicht hinhaut?" (Hafenlotsen)*

	**Überregionaler Austausch zwischen Akteuren**	*„Ja, durchaus, das ist immer wichtig. Das kennt man ja aus der Seefahrt Manöverkritik praktisch, also man würde das Ganze noch mal bewerten, gegebenenfalls natürlich auch Schlüsse daraus ziehen, was man beim nächsten Mal anders machen würde. Ganz klar. Und natürlich ist auch in dem Zusammenhang vielleicht noch mal als Anregung wichtig, ein Austausch vielleicht mit anderen Bundesländern und anderen Häfen, dass man dort sich auf einer gemeinsamen Plattform trifft und sich austauschen würde in diesen Punkten. Das würde ich sehr begrüßen." (Hafenkapitän)*<br><br>*„Ein Infektionsfall in Hamburg, ist nicht anders als in Bremerhaven. […] ich hätte durchaus ein Interesse daran, dass so etwas vielleicht auf einer bestimmten Ebene gehoben wird, wenn man sagt, man hat ein jährliches Treffen, wo man drüber spricht, wo man eine Expertenkommission einsetzt, die Empfehlungen raus gibt oder so. Das kann einen nur unterstützen, weil man muss deutlich sagen, es ist ein sehr seltenes Ereignis, es fällt oft runter im Alltag, das muss man deutlich sagen, das fällt oft runter. Und die personelle Besetzung ist nun bei den Dienststellen, und da spreche ich wohl für alle Hafenstädte, nicht so, dass man durchdekliniert, das mache ich jetzt morgen, mal überlegen, ich könnte mich mal damit befassen, sondern eigentlich haben wir immer ein Berg, den man irgendwie versucht abzuarbeiten. Und wenn man dann eine Institution oder eine Gruppe oder jemand Motiviertes hat der sagt "ich kümmere mich mal darum und hole die alle zusammen, und wir sprechen dann da drüber" dann haben wir als Feuerwehr [Ort] dann sehr großes Interesse, auch daran teilzunehmen und diese Empfehlungen dann auch in unsere Einsatzplanung einfließen zu lassen." (Feuerwehr)*<br><br>*„Also hier bei der Einarbeitung: Infektionsschutz, das ist natürlich ein ganz großes Thema. Also ich denke, man könnte auch überlegen, dass man so ein Hospitationsforum anbietet. Also ich würde sagen, wir haben relativ viele infektiöse Fälle hier. Aber man weiß ja, dass die kommen, halt ohne Ankündigung. Es kann eine Woche, zwei, drei, vier vergehen, das nichts ist, und dann ist doch was. Aber da könnte man natürlich mal überlegen, ob Kollegen aus kleineren Häfen hospitieren oder so. Das wäre vielleicht etwas. Ich weiß zwar nicht, wie man dann, ohne echten Fall, müsste man es halt einfach nur simulieren dann." (Hafenärztlicher Dienst)*
**Oberkategorie Schnittstellen und Informationsbedarf im Einsatz**	**Informationsübermittlung**	*„Ja, Information ist ja schon mal ich sag mal das "a und o", dass man weiß, wie es ist, und ich sag mal, wenn dann auch drinsteht, ich sage jetzt mal, der oder die betroffene Person ist separiert. Das beruhigt ja dann auch die Kollegen und vor allen Dingen, wenn man auch noch mal weiß, wenn es einer aus der Maschine ist, muss ich mir keine Gedanken machen, dass da oben an der Brücke die ganze Zeit sich aufgehalten hat. Wenn es ein nautischer Offizier ist, wird die Sache natürlich dann schon interessanter." (Kanalsteuerer)*<br><br>*„Ja, ja, man schafft es nur durch vernünftige Informationen, transparent viel informieren."* (Wasserschutzpolizei)<br><br>*Wir haben ja einen 24/7 Wachdienst. Wenn die Schiffe sich anmelden und sagen, dass sie bunkern wollen oder Wasser brauchen und dann schon die Information vorhanden wäre, dass darauf ein Infektionsfall sein könnte, das wäre schon viel wert. (Lotsen)*
	**Lageeinschätzung**	*„Also ich glaube, ein Fall gab es wohl, dann sollte ein Arzt, sollte die Besatzung, wie sagt man, begutachten, checken, und der sagt, ne, ich fahr da nicht, ich gehe dann die Leiter nicht hoch. Kann ich verstehen, ist ja nicht jedermanns Sache, aber es wäre vielleicht manchmal dann der einfachere Weg. Aber ich muss nicht erst das ganze Schiff an die Pier bringen, damit der Arzt da irgendwie dann an Bord gehen kann. Vielleicht hält man Leute vor, die dann da auch durchaus in der Lage sind, da mal eine Treppe und Leiter hochzugehen, um eine Einschätzung zu machen. Ist glaube ich einmal passiert." (Hafenlotsen)*
	**Meldeketten**	*„Ja, diese Verbindlichkeit der Meldeketten, wenn die noch besser ausgebaut werden würde, […], es waren aber auch dann in den Verteilern immer nochmal wieder alte Leute drin. Also das ist noch nicht richtig. Also da war dann wieder, wir hatten ja gerade auch den Wechsel […], und bis wir dann alle drin waren, dann hat der andere die Mail bekommen, und hier ging sie zweimal hin und da mal nicht. Also, das war alles irgendwie ein bisschen zusammengestrickt, und das könnte man bestimmt noch deutlich verbessern, dass man da klare Meldewege hat." (Hafenlotsen)*
**Oberkategorie Digitalisierung**	**Melde- und Alarmsysteme**	*„Digitalisieren, digitalisieren, zum Beispiel Paragraph 12 Meldungen, also eben der GNIT-Fall, ist im Infektionsschutzgesetz in Paragraph zwölf noch mal abgelichtet. Ist ein Papierformular als Fax oder als E-Mail. Es wäre natürlich gut, wenn das digital laufen würde, zum Beispiel, wenn ich das an unsere deutsche RKI Stelle übersende und schade, dass sowas nicht über Surfnet oder Demis läuft. Generell wie Infektionskrankheiten gemeldet werden. Dass das läuft da einfach noch ein bisschen in Steinzeitprozedere." (Hafenärztlicher Dienst)*<br><br>*„Ja, na ja, wir reden ja von Digitalisierung. Das wäre schon sehr großes Thema. Da hängt Deutschland auch, glaube ich, weit hinterher. Da habe ich auch ein lustiges Beispiel, aber das ein ganz anderes Thema. Dass es irgendwelche elektronischen Meldesysteme oder Meldemöglichkeiten geben könnte, dass man eben auch dann, gut ich kann natürlich, der kann mir eine Mail schreiben und dann lese ich die erst nächsten Morgen. Es gibt aber Mails, die wichtig sind, die man sofort lesen müsste, dass dann irgendwas irgendwo anfängt zu piepen oder keine Ahnung, das ich weiß, und wenn es nur ein, zwei Mal im Jahr ist oder einmal in zehn Jahren, aber dann, das ist schon, ist ja wichtig, dass ich dann eben sehe, okay, was ist da los, was will er? Am Ende des Tages, ich kann digitalisieren so viel will ich will, irgendein Roboter oder ein digitales System kann das Schiff da nicht hinlegen oder, na gut Ich weiß nicht, ob es Roboter gibt die impfen können oder einen Knochenbruch schienen, ich weiß es nicht, aber irgendwann geht's müssen dann Menschen da sein, die agieren, am Ende des Tages. Einer, der sich ins Auto setzt, Motor anmacht und losfährt, um irgendjemand abzuholen. Das muss dann am Ende doch sein. Aber wie gesagt, möglichst sichere elektronische Meldemöglichkeit wäre schon gut." (Hafenbehörde)*
	**Aussteigerkarten**	*„Dasselbe Spiel mit der Aussteigerkarte auf dem Kreuzfahrtschiff. Wenn die Passagiere dann im Rahmen von Großschadensereignissen aussteigen, geben die eine Aussteigerkarten ab, und dies halt auch in Papierform. Das alles dann durchzugehen. Wer hatte Kontakt? Gibt es weitere Kontaktperson, und welche weiteren Kreise, Städte, Länder müssen wir informieren? Wenn da 5000 Leute sind, dann hast du da so ein Werk, was natürlich, wenn das digital wäre, kannst du sofort Filter setzen, und dann hast du sofort das, was du da brauchst. Sowas wäre natürlich gut für die Zukunft." (Hafenärztlicher Dienst)*
	**Ampelsystem**	*„Sowas würde uns helfen. Das hat aber Rostock. […] Wir haben ja immer das Problem, Stichwort Durchgangsrevier. Das heißt, wir haben relativ häufig wechselnde Schiffe. Insofern ja, wäre sicherlich ein richtiger Schritt, wenn man weiß, okay, da ist schon mal was rot oder grün oder gelb auf der Ampel. Nun weiß ich aber natürlich auch, was würde ich denn machen als Kapitän, der weiß da unten ist einer, der hat irgendwas und der Charterer sitzt mir im Nacken. Und eigentlich will ich im nächsten Hafen sowieso Abmustern und nach Hause fliegen. Ja, auf die Ehrlichkeit des Kapitäns ist man halt in dem Fall darauf angewiesen. Also ich bin da immer so ein bisschen kritisch von diesen ganzen Erklärungen, weil nirgendwo so so viel gelogen wo Dokumentation stattfindet." (Hafenlotsen)*
	**Plattform zur Datenübertragung**	*„Ich denke, es wäre gut, wenn wir eine gemeinsame Plattform hätten, auf der wir mit der Hafenbehörde über Kurven, Zahlen und Maßnahmen sprechen könnten, denn es ist natürlich gut, zu telefonieren. Aber auch aus der Perspektive der epidemiologischen Studien und des MBA haben wir uns wirklich damit beschäftigt, und es ist so interessant, wie wir, die wir keine Konkurrenten sind, das gleiche Ziel verfolgen, aber selbst Konkurrenten Daten austauschen, wenn es in ihrem eigenen Interesse ist. Und ich denke, wir alle müssen dabei gewinnen. Und ich hätte nichts dagegen, wenn sie Zugang zu unseren Daten hätten, denn ich glaube, das würde den Gesprächsprozess wirklich beschleunigen. Wenn es ein Gespräch gibt oder wenn sie einfach nur glücklich sind, würde es ihnen einige Kopfschmerzen ersparen, zum Schiff zu gehen und zu versuchen, zu verstehen, was passiert ist. Sie würden einfach hingehen und bereits wissen, was passiert und nur stichprobenartig überprüfen, was sie überprüfen müssen. Und ich denke, das ist die Zukunft." (Reederei)*
	**Stabsarbeitssoftware**	*„Es gibt ja kaum gemeinsame Plattformen, über die man / oder also es gibt nichts Vorgeplantes. Das heißt klassisch Telefon, eMail und natürlich dann mit gewissen Einheiten über Funk. Aber deswegen fand ich das ARMIHN Projekt so toll. Da gab es ja diese eigene Plattform, die man da konstruiert hat. Die war echt genial, aber leider haben wir da nichts. Ja, das war eine Plattform, wo Informationen verbreitet werden konnten, über die alle Akteure bei der Übung sich einklinken konnten und wo dann verschiedene, auch Sachen hochgeladen werden konnten, im Grunde eine Datenaustauschplattform. Nichts Großartiges. Aber dass das System, dass es das überhaupt gab und gut gelaufen ist, das war eine schöne Sache, aber leider ansonsten halt, wie gesagt, Telefon, eMail, das ist der klassische Kommunikationsweg." (Havariekommando)*

 © Thieme. All rights reserved.
